# Comparison of UTCI with Other Thermal Indices in the Assessment of Heat and Cold Effects on Cardiovascular Mortality in the Czech Republic

**DOI:** 10.3390/ijerph110100952

**Published:** 2014-01-09

**Authors:** Aleš Urban, Jan Kyselý

**Affiliations:** 1 Institute of Atmospheric Physics AS CR, Boční II 1401, 141 31 Prague 4, Czech Republic; E-Mail: kysely@ufa.cas.cz; 2 Department of Physical Geography and Geoecology, Faculty of Science, Charles University, Albertov 6, 128 43 Prague 2, Czech Republic

**Keywords:** UTCI, human thermal comfort, mortality, cardiovascular diseases, heat stress, cold stress

## Abstract

We compare the recently developed Universal Thermal Climate Index (UTCI) with other thermal indices in analysing heat- and cold-related effects on cardiovascular (CVD) mortality in two different (urban and rural) regions in the Czech Republic during the 16-year period from 1994–2009. Excess mortality is represented by the number of deaths above expected daily values, the latter being adjusted for long-term changes, annual and weekly cycles, and epidemics of influenza/acute respiratory infections. Air temperature, UTCI, Apparent Temperature (AT) and Physiologically Equivalent Temperature (PET) are applied to identify days with heat and cold stress. We found similar heat effects on CVD mortality for air temperature and the examined thermal indices. Responses of CVD mortality to cold effects as characterised by different indices were much more varied. Particularly important is the finding that air temperature provides a weak cold effect in comparison with the thermal indices in both regions, so its application—still widespread in epidemiological studies—may underestimate the magnitude of cold-related mortality. These findings are important when possible climate change effects on heat- and cold-related mortality are estimated. AT and PET appear to be more universal predictors of heat- and cold- related mortality than UTCI when both urban and rural environments are of concern. UTCI tends to select windy rather than freezing days in winter, though these show little effect on mortality in the urban population. By contrast, significant cold-related mortality in the rural region if UTCI is used shows potential for UTCI to become a useful tool in cold exposure assessments.

## 1. Introduction

An adverse effect of heat and cold stress on mortality due to cardiovascular diseases has been reported in many studies [1]. Most of these employed air temperature or another simple measure of equivalent temperature (empirical indices) including effects of air temperature, humidity and/or wind speed (apparent temperature, heat index *etc.*, [2]). Human thermal comfort is an outcome of energy balance between the human body surface and the environment, and it is influenced by human physiology, psychology and behaviour [3,4]. Not all of these factors are well characterized by empirical indices, which, therefore, are unable to serve all human-biometeorological applications (e.g., public weather service, public health system, urban and regional planning, climate impact in the health sector) across all climatic zones, regions and seasons [4].

Human thermal comfort models, on the other hand, consider in addition to atmospheric parameters (air temperature, water vapour pressure, wind speed and mean radiant temperature [5]) complex metabolic processes including physical activity level and clothing insulation [4]. Human thermal comfort indices such as Physiologically Equivalent Temperature (PET [6,7,8]) based on the Munich Energy—Balance Model for Individuals (MEMI) and the Klima–Michel model with Perceived Temperature (PT) as the equivalent temperature [9,10] have been commonly used in human-biometeorological assessments during the last decade [11,12,13,14,15,16]. One of the most advanced models based on the latest progress in all associated disciplines (thermal physiology, occupational medicine, physics, meteorology, as well as biometeorological and environmental sciences) is the Fiala multi-node model of human thermoregulation [17] with a derived equivalent temperature Universal Thermal Climate Index (UTCI). It has been developed in order to create a standard measure for outdoor thermal conditions suitable in all major fields of human biometeorology [4,18]. The Fiala thermophysiological model is coupled with a clothing model which defines in detail the effect of clothing insulation for each of the body segments over a wide range of climatic conditions [19]. UTCI, in comparison with other indices, is more sensitive to even slight changes in temperature, solar radiation, humidity and wind speed and describes better various climatic conditions, which might be an opportunity for more appropriate human-biometeorological assessments [20].

Błażejczyk [20] compared UTCI with other indices using meteorological data. Only a few studies, however, have evaluated how UTCI performs compared to other indices in assessing epidemiological outcomes [13,16], and their results have been unconvincing. Indeed, no significant predictive advantage for any thermal index (including UTCI) over the use of air temperature has been observed. Both aforementioned studies [13,16] were carried out in a warm climate (Bangladesh and Greece), and there have been no studies comparing the applicability of various human thermal comfort indices for studying heat- and cold-related mortality in populations living under temperate climatic conditions (such as central Europe).

Recent epidemiological data available in the Czech Republic allow for more detailed study of heat and cold stress impacts on individual cardiovascular (CVD) diagnoses (groups of diagnoses) and enable the study of regional differences within the country. This paper resumes our earlier research [21] and investigates differences in heat- and cold-related cardiovascular mortality evaluated in terms of different thermal indices in an urban and a rural region in the Czech Republic. In the context of a complex thermal environment, we compared UTCI with other thermal indices and with air temperature for their abilities to identify days with adverse thermal conditions for persons with cardiovascular diseases. A special focus was given to differences in the performance of various indices under cold stress conditions due to the effect of wind. 

## 2. Data and Methods

**Figure 1:**
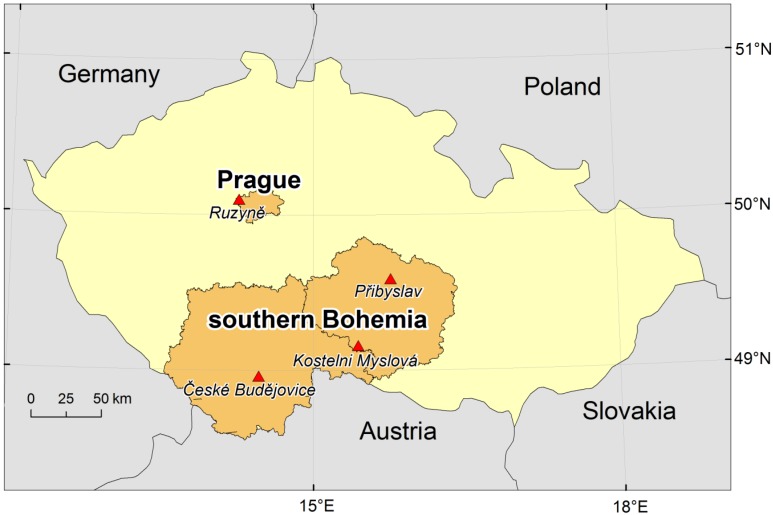
Study areas. Red triangles show the meteorological stations used.

Daily data on mortality due to CVDs (codes I00–I99 according to the International Statistical Classification of Diseases, 10th Revision [ICD-10]), covering the period 1994–2009, were provided by the Czech Statistical Office (CZSO) and the Institute of Health Information and Statistics (IHIS). The data were sorted according to the primary cause of death (Table 1) and region of residence. Two regions with different characteristics—the city of Prague (1.25 million inhabitants) and the southern Bohemian region (1.15 million inhabitants)—were defined as urban and rural regions in accordance with the OECD’s international definition [22,23]. OECD’s terminology [22] defines a rural region as one in which at least 37.5% of inhabitants live in municipalities with population density less than 150 inhabitants per km^2^. In accordance with this definition, the two regional administrative units in the Czech Republic with the largest proportions of rural population are the South Bohemia Region (Jihočeský kraj, 46.8%) and the adjoining Highlands Region (Kraj Vysočina, 51.9%) [23]. Together, these constitute a contiguous geographic region (southern Bohemia; Figure 1) with population size and structure similar to Prague, but only 36% of inhabitants live in municipalities with population above 10,000 [24]. More details about the population under study are given in [21].

**Table 1:** Examined diagnoses according to the International Statistical Classification of Diseases (ICD–10) coding and abbreviations used.

ICD-10 code	Abbreviation	Diagnosis
I00–I99	CVD	cardiovascular disease
I20–I25	IHD	ischemic heart disease
I60–I69	CD	cerebrovascular disease
I21–I22	MI	myocardial infarction (acute and subsequent)
I25	CIHD	chronic ischemic heart disease
I70	ASVD	atherosclerosis – atherosclerotic vascular disease

An indirect standardization procedure, analogous to that in [25,26], was used to adjust mortality data for long-term changes, as well as seasonal and weekly variations. The expected number of deaths for every day of the examined period *M_0_*(*y*,*d*) for year *y* (*y* = 1994, ... 2009) and day *d* (*d* = 1, ... 365) was determined according to the formula:

*M_0_*(*y*,*d*) = *M_0_*(*d*).*W*(*y*,*d*).*Y*(*y*)

In the equation, *M_0_*(*d*) denotes the mean daily mortality on day *d* in a year (computed from the mean annual cycle over 1994–2009). In view of known relationships between influenza/acute respiratory infections (ARI) and CVD mortality [27,28], 169 winter days during six epidemics were omitted from the analysis (before calculating the mean annual cycle) in order not to confound results (see also [21]). *W*(*y*,*d*) is a correction factor for the observed weekly cycle of mortality, calculated separately for individual days of the week and defined as the ratio of the mean mortality on a given day to the overall mean mortality, and *Y*(*y*) is a correction factor for the observed year-to-year changes in mortality, defined as the ratio of the number of deaths in year *y* to the mean annual number of deaths during the analyzed period. The correction factors for the weekly cycle *W*(*y*,*d*) and the year-to-year changes *Y*(*y*) were calculated over the April–November period when epidemics of influenza/ARI did not occur. When calculating *W*(*y*,*d*), all public holidays were excluded, too. An output of the standardization procedure is an expected number of deaths for every day of the examined period (*baseline mortality*), and deviations of observed and expected mortality determine *excess mortality*. Relative deviations (in %) from the baseline mortality are presented in results.

Because of the need for more input variables, different meteorological datasets were used in comparison to those from our previous study [14]. Data on air temperature (*T*, in °C), wind speed at 10 m above surface (*v_10_*, m∙s^−1^), relative humidity (*RH*, %) and cloudiness (*C*, octas) from the Prague-Ruzyně (airport) station and three southern Bohemian stations (České Budějovice, Kostelní Myslová, Přibyslav), were obtained from the Czech Hydrometeorological Institute (CHMI; Figure 1). All stations measured three times daily in standard climatic terms (7:00, 14:00 and 21:00 local time) and covered the same period of 1994–2009.

The heat budget-based indices—PET and UTCI—were calculated from air temperature, relative humidity, wind speed, and mean radiant temperature (Tmrt) that was modelled as a function of air temperature and cloudiness, in the RayMan Pro model (Version 2.1) [29,30]. For the calculation of PET, it is necessary to consider meteorological input parameters important for the human energy balance at a height relevant for human-biometeorological assessment [6,7]. Therefore, the wind speed was recalculated to the height of 1.1 m above surface, using Hellman’s exponential law [31]:

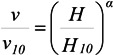

where *v* is the wind speed at height *H* = 1.1 m, *v_10_* is the wind speed at height *H_10_* = 10 m, and *α* is the friction coefficient (Hellman exponent). We used *α* = 0.40 for the urban area (Prague) and *α* = 0.30 for the rural area according to Table 1 in [31]. PET is then defined as an equivalent temperature in a typical indoor setting (without wind and solar radiation) at which the energy balance of the sitting reference person (with the same core and skin temperature) is equal to that under the actual outdoor conditions to be assessed [7]. We note that the reference height at which wind speed is considered when calculating PET is important for the PET values but has little influence on the selection of warm/cold days (above/below the 90%/10% quantile) in our study, as the samples of warm and cold days were very similar if PET was calculated from wind speed at 10-m height.

UTCI is an equivalent temperature defined for a walking person (4 km/h) with adaptive clothing [19] in referent outdoor conditions with 50% relative humidity, still air, and Tmrt equalling air temperature [4]. Wind speed at 10-m height is used for the UTCI calculation (by definition) [32]. Apparent Temperature (AT) is the temperature at the reference humidity level requiring the same thermal resistance of a walking adult as that experienced under the current ambient temperature, humidity, wind and solar radiation [33]. The Steadman’s “non-radiation” formula [34,35] was used for the AT calculation: AT = *T* + 0.33∙*vp* − 0.7∙*v_10_* − 4.0. Since it includes in addition to vapour pressure (*vp*) also the “wind chill” effect of wind speed (*v_10_*), AT is applicable in a wide range of temperatures. Vapour pressure values were calculated from air temperature and humidity in the RayMan Pro model. All indices were computed separately for every station at each observation time (7:00, 14:00 and 21:00 local time) and then averaged to obtain mean daily values.

Days with mean (equivalent) temperature (T, AT, PET, and UTCI) above/below the 90%/10% quantile of the empirical distribution in summer (June–August)/winter (December–February (in the next year)) seasons over 1994–2009 were defined as *warm*/*cold* days. Use of the percentile method (unlike determining an exact temperature threshold) allows for examining approximately the same sample sizes in different regions and at both temperature extremes. This method has commonly been used for regional comparison of heat and cold impact on human health [14,36,37,38]. Because some winter days were omitted due to influenza/ARI epidemics, and also because winter seasons (usually 90 days) are shorter than summer seasons (92 days), the examined samples of cold days are slightly smaller than those of warm days.

Summed and averaged deviations from the expected values of mortality over all warm/cold days (D_0_) and one day thereafter (D_+1_, to capture basic lagged effects) were calculated. In the case of consecutive warm/cold days, only D_0_ values were included into the calculation so that no day is counted twice. The deviations’ significance was evaluated using 95% confidence intervals (CI), calculated using the limit factors for a Poisson-distributed variable according to [39]. When the number of cases was larger than 100, the normal approximation was used.

## 3. Results

**Figure 2:**
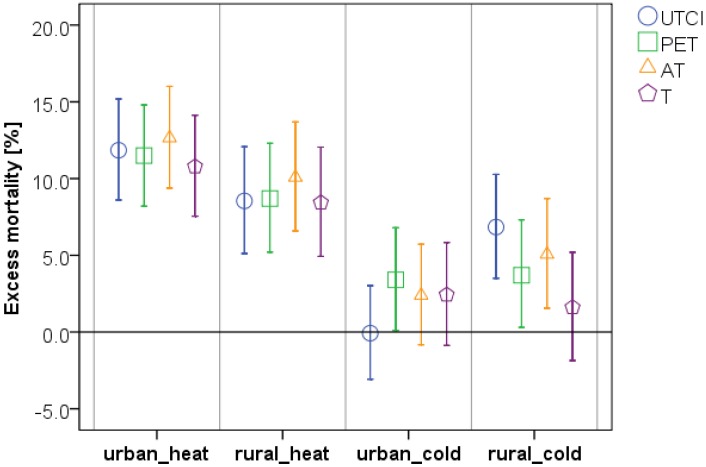
Mean relative excess CVD mortality (% above the expected value) for warm and cold days as determined by individual indices in the urban *versus* rural region. Error bars represent the 95% CI (specific values are given in Table 2 and Table 5).

On warm days in both regions, excess mortality tends to be highest when AT is used to determine those days (Figure 2). This holds true for all examined groups of diagnoses except for atherosclerosis (ASVD) (Table 2). We found similar urban–rural differences for all indices, with slightly higher mortality deviations in the urban region. This pattern holds true also for most examined groups of diagnoses.

**Figure 3:**
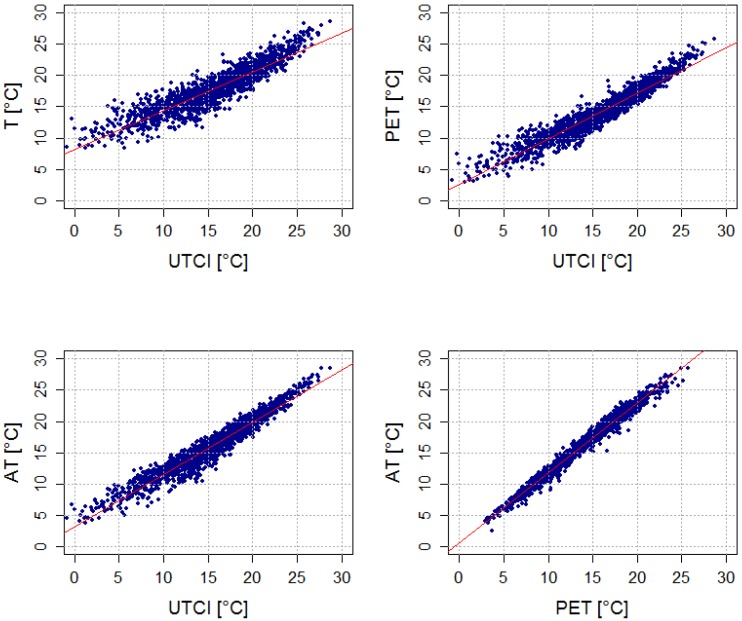
Linear regression between mean daily UTCI, PET, AT, and T in the urban (Prague) region in summer over 1994–2009. Coefficients of determination are shown in Table 3.

All indices fit well with one another in summer (Table 3, Figure 3). PET and AT fit equally well with UTCI (the coefficient of determination (R^2^) is around 0.93, and there are about 83% warm days in common for these pairs of indices in both regions), although the AT calculation does not include the effect of Tmrt. The relationship between air temperature and UTCI is weaker (about 75% warm days in common). The average Tmrt values were very similar for all indices on warm days, however, and the other input meteorological variables (T, *v_10_*, RH, C) also showed few differences in the two regions (Table 4).

**Table 2:** Relative excess cardiovascular mortality with 95% CI (in parentheses) on warm days, defined as days with average (equivalent) temperature above the 90% quantile of the empirical distribution (≥°C), in Prague and southern Bohemia over 1994–2009. Values significantly different from zero are highlighted in bold.

**Urban Region (Prague)**				
Thermal index/Diagnosis	UTCI (≥22.0 °C)	PET (≥19.2 °C)	AT (≥22.0 °C)	T (≥22.5 °C)
CVD	**11.8 (8.6; 15.2)**	**11.5 (8.2; 14.8)**	**12.6 (9.4; 16.0)**	**10.8 (7.5; 14.1)**
IHD	**7.8 (2.9; 13.0)**	**8.0 (3.0; 13.2)**	**8.7 (3.7; 13.9)**	**7.0 (2.1; 12.2)**
CD	**11.6 (5.4; 18.2)**	**9.5 (3.3; 16.1)**	**13.0 (6.7; 19.7)**	**10.0 (3.8; 16.6)**
MI	−0.8 (−8.8; 7.9)	1.5 (−6.6; 10.4)	−0.2 (−8.2; 8.5)	−1.6 (−9.6; 7.1)
CIHD	**12.0 (5.8; 18.6)**	**10.9 (4.7; 17.5)**	**13.0 (6.8; 19.7)**	**10.9 (4.7; 17.4)**
ASVD	**17.6 (10.0; 25.7)**	**18.5 (10.9; 26.6)**	**18.5 (10.9; 26.5)**	**19.4 (11.6; 27.7)**
**Rural Region (Southern Bohemia)**				
Themal index/Diagnosis	UTCI (≥21.9 °C)	PET (≥18.0 °C)	AT (≥21.7 °C)	T (≥22.0 °C)
CVD	**8.5 (5.1; 12.1)**	**8.7 (5.2; 12.3)**	**10.1 (6.6; 13.7)**	**8.4 (4.9; 12.0)**
IHD	**6.9 (2.0; 12.0)**	**7.3 (2.3; 12.5)**	**7.9 (2.9; 13.2)**	**7.2 (2.2; 12.5)**
CD	**11.4 (5.0; 18.3)**	**10.3 (3.8; 17.2)**	**13.5 (6.9; 20.6)**	**7.9 (1.3; 14.8)**
MI	1.6 (−5.6; 9.4)	1.2 (−6.1; 9.1)	2.0 (−5.4; 9.9)	3.6 (−3.9; 11.8)
CIHD	**11.4 (4.8; 18.4)**	**11.9 (5.2; 19.0)**	**12.4 (5.6; 19.5)**	**10.2 (3.5; 17.4)**
ASVD	**9.8 (0.4; 20.2)**	**10.9 (1.4; 21.3)**	**11.2 (1.5; 21.8)**	**14.9 (5.0; 25.6)**

**Table 3:** Coefficients of determination (R^2^) for linear relationships between mean daily UTCI, PET, AT, and T in the urban *vs*. rural region in summer (JJA) and winter (DJF) seasons. (%) denotes percentages of warm (cold) days in common in summer (winter) during 1994–2009.

Thermal Indices Compared	Urban JJA R^2^ (%)		Urban DJF R^2^ (%)		Rural JJA R^2^ (%)		Rural DJF R^2^ (%)	
T~UTCI	0.83	73	0.23	31	0.86	77	0.52	45
PET~UTCI	0.91	82	0.41	39	0.92	82	0.68	59
AT~UTCI	0.93	83	0.56	45	0.94	84	0.75	62
T~AT	0.94	79	0.88	86	0.95	84	0.94	81
AT~PET	0.98	82	0.95	91	0.98	87	0.98	91
T~PET	0.96	84	0.95	88	0.95	87	0.96	80

**Table 4:** Average values of (equivalent) temperature indices and input meteorological variables on warm days identified by individual indices in urban *vs*. rural region.

**Urban Region**	**UTCI (°C)**	**PET (°C)**	**AT (°C)**	**T (°C)**	**Tmrt (°C)**	**v*_10_* (m∙s^−1^)**	**RH (%)**	**C (octas)**
UTCI	23.9	20.8	23.7	23.8	29.7	2.3	59	3.7
PET	23.7	21.0	23.7	24.0	29.8	2.6	58	3.9
AT	23.8	20.8	23.8	24.0	29.6	2.5	60	3.5
T	23.4	20.8	23.5	24.2	29.8	2.9	56	3.3
**Rural Region**	**UTCI (°C)**	**PET(°C)**	**AT (°C)**	**T (°C)**	**Tmrt (°C)**	**v*_10_* (m∙s^−1^)**	**RH (%)**	**C (octas)**
UTCI	23.4	19.3	23.2	23.2	28.8	2.2	62	2.6
PET	23.2	19.4	23.2	23.3	28.7	2.4	61	2.6
AT	23.3	19.4	23.3	23.3	28.6	2.4	63	2.5
T	23.1	19.3	23.2	23.5	28.9	2.6	60	2.4

On cold days, by contrast, we found no general pattern of higher excess mortality for any index (Figure 2) and the results depend on region and diagnosis (Table 5). The urban–rural differences in cold-related mortality for individual indices are much less consistent compared to heat-related mortality. While mean excess mortality is comparable (predominantly insignificant differences) for PET, AT and T in Prague, it is higher and mostly significant for UTCI and AT in southern Bohemia. In particular, UTCI indicates substantial difference between no cold effect (−0.1%, 95% confidence interval (CI) −3.1% to 3.0%) on CVD mortality in Prague but significant 6.8% (3.5% to 10.3%) excess mortality in southern Bohemia, a pattern that is not found for any other index.

**Figure 4:**
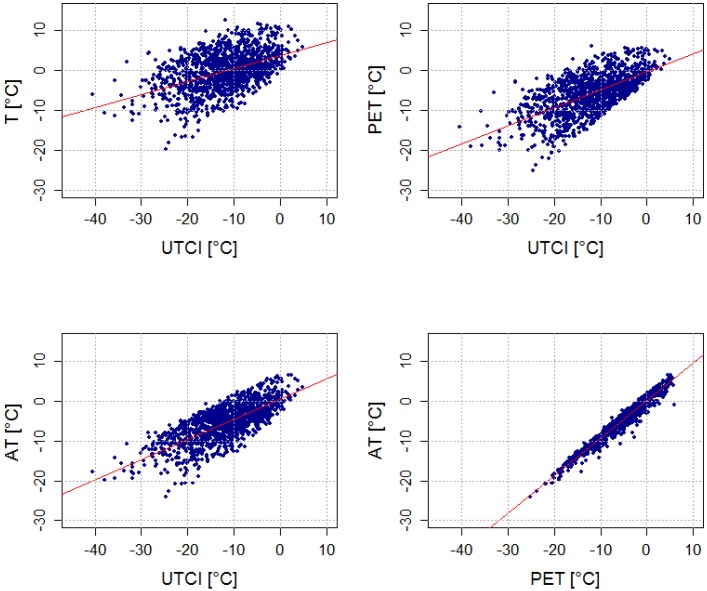
Linear regression between mean daily UTCI, PET, AT, and T in the urban (Prague) region in winter over 1994–2009. Coefficients of determination are shown in Table 3.

UTCI correlates weakly with the other indices and air temperature in winter (Table 3, Figure 4), while PET and AT are much more strongly linked to air temperature. Moreover, little consensus in the selection of cold days between UTCI and the other indices was found in the two regions. In Prague, only 31% of cold days were common for air temperature and UTCI, and the R^2^ for all winter daily values of T and UTCI was just 0.23.

Examining specific diagnoses and groups of diagnoses, the general pattern of heat-related mortality was associated primarily with chronic CVDs (atherosclerosis (ASVD), chronic ischemic heart disease (CIHD)) while the highest cold-related mortality from acute myocardial infarction (MI) observed in both regions and for all indices was in agreement with our previous study based on air temperature only [21]. However, while the heat-related mortality deviations show a similar pattern of differences between individual diagnoses (with the largest deviations for ASVD and the lowest for ischemic heart disease (IHD) and MI) for all indices (Table 2), the differences in excess mortality for individual groups of diagnoses on cold days are much less consistent (Table 5). UTCI and AT indicate significant (*p* = 0.05) excess mortality in the rural region also for both main subgroups of CVDs (IHD and cerebrovascular disease (CD)). This is in contrast with the results for air temperature, in relation to which excess cold-related mortality is very small in both regions and all diagnoses, except for MI.

**Table 5:** Relative excess cardiovascular mortality with 95% CI (in parentheses) on cold days, defined as days with average (equivalent) temperature above the 90% quantile of the empirical distribution (≤°C), in Prague and southern Bohemia over 1994–2009. Values significantly different from zero are highlighted in bold.

**Urban Region (Prague)**				
Thermal index/Diagnosis	UTCI (≤−21.5 °C)	PET (≤−12.9 °C)	AT (≤−12.1 °C)	T (≤−6.5 °C)
CVD	−0.1 (−3.1; 3.0)	**3.4 (0.1; 6.8)**	2.4 (−0.8; 5.7)	2.4 (−0.9; 5.8)
IHD	−0.6 (−5.4; 4.3)	3.9 (−1.4; 9.4)	3.8 (−1.3; 9.3)	2.1 (−3.1; 7.6)
CD	0.9 (−5.0; 7.2)	4.6 (−2.0; 11.5)	4.1 (−2.3; 11.0)	4.6 (−2.0; 11.6)
MI	4.5 (−3.6; 13.3)	**10.6 (1.3; 20.7)**	**10.5 (1.4; 20.4)**	7.1 (−2.1; 17.1)
CIHD	−3.1 (−8.9; 3.0)	0.6 (−5.7; 7.3)	0.5 (−5.8; 7.1)	−0.3 (−6.7; 6.5)
ASVD	1.7 (−4.7; 8.5)	3.6 (−3.4; 11.1)	1.4 (−5.2; 8.5)	2.8 (−4.0; 10.1)
**Rural Region (Southern Bohemia)**				
Thermal index/Diagnosis	UTCI (≤−19.9 °C)	PET (≤−13.5 °C)	AT (≤−12.4 °C)	T (≤−7.3 °C)
CVD	**6.8 (3.5; 10.3)**	**3.7 (0.3; 7.3)**	**5.1 (1.6; 8.7)**	1.6 (−1.9; 5.2)
IHD	**8.3 (3.4; 13.5)**	3.3 (−1.8; 8.5)	**5.7 (0.6; 11.1)**	2.0 (−3.1; 7.4)
CD	**10.7 (4.4; 17.3)**	**7.0 (0.5; 14.0)**	**8.0 (1.4; 15.1)**	2.7 (−3.8; 9.5)
MI	**15.2 (7.4; 23.6)**	**12.6 (4.4; 21.4)**	**17.2 (8.8; 26.2)**	**10.6 (2.4; 19.5)**
CIHD	2.9 (−3.4; 9.6)	−3.5 (−9.8; 3.2)	−2.7 (−9.1; 4.2)	−4.3 (−10.7; 2.6)
ASVD	−1.5 (−10.3; 8.2)	−1.4 (−10.7; 8.8)	−1.7 (−11.1; 8.7)	1.5 (−7.9; 11.9)

## 4. Discussion

In this study, we tested the extent to which UTCI, other thermal indices (PET, AT), and air temperature are able to identify days with adverse thermal conditions for persons with cardiovascular diseases (CVD). While similar heat effects for air temperature and thermal indices on cardiovascular (CVD) mortality were found in both urban and rural region of the Czech Republic, we observed no general pattern of higher excess mortality for any index on cold days and the urban-rural differences in cold-related mortality were much less consistent compared to heat-related mortality. In particular, UTCI indicates substantial difference between no cold effect on CVD mortality in Prague but significant excess mortality in southern Bohemia.

The different cold effects of air temperature and thermal indices are related to the different samples of cold days for individual indices (Table 6). The enhanced cooling effect of wind involved in the UTCI calculation [20] is probably the main reason for this dissimilarity. While the average wind speed at Prague-Ruzyně airport on cold days is approximately twice as high for UTCI (7.2 m∙s^−1^) in comparison to the other indices (2.6–3.4 m∙s^−1^), average air temperature is higher as well (−4.4 °C *vs*. −9.3 to −9.6 °C; Table 6). Also the other meteorological variables, in particular Tmrt, show substantially different average values on cold days as defined by UTCI in comparison to those defined by the other indices. A similar pattern exists in the rural area.

**Table 6:** Average values of (equivalent) temperature indices and input meteorological variables on cold days identified by individual indices in urban *versus* rural region.

**Urban Region**	**UTCI (°C)**	**PET (°C)**	**AT (°C)**	**T (°C)**	**Tmrt (°C)**	**v*_10_* (m∙s^−1^)**	**RH (%)**	**C (octas)**
UTCI	−25.5	−11.5	−12.2	−4.4	−9.0	7.2	81	5.7
PET	−20.9	−15.7	−14.7	−9.3	−16.3	3.2	83	4.0
AT	−21.4	−15.6	−14.8	−9.3	−16.0	3.4	83	4.3
T	−19.3	−15.5	−14.6	−9.6	−16.3	2.6	84	4.3
**Rural Region**	**UTCI (°C)**	**PET (°C)**	**AT (°C)**	**T (°C)**	**Tmrt (°C)**	**v*_10_* (m∙s^−1^)**	**RH (%)**	**C (octas)**
UTCI	−24.4	−14.1	−13.6	−6.8	−12.1	5.5	82	5.5
PET	−21.8	−16.1	−15.0	−9.4	−15.9	3.5	82	4.2
AT	−22.2	−16.0	−15.1	−9.4	−15.7	3.7	82	4.4
T	−19.6	−15.8	−14.7	−9.6	−16.3	2.8	82	3.9

The rapid fall in UTCI due to wind speed in cold weather has been documented by Novák [40]. UTCI selects windy rather than freezing days in winter, and these show little effect on excess mortality among the urban population that is well protected against wind. However, windy winter weather with not extremely low air temperatures (but still far below 0 °C) may be related to more snowfalls [41] and therefore to diminished accessibility of small villages in the rural region, where the cold effect on mortality is most pronounced as indicated by UTCI. An analysis of relationships between cold days and the snowfalls in the two regions may help to explain the observed pattern, as there has been some evidence of a relationship between snowy weather and higher cardiovascular mortality [42]. This relates, among other things, to physical exertion due to snow shovelling [43,44].

Our results extend previous findings [13,15,16,45] that air temperature (T), as the most widely used proxy for ambient thermal conditions in environmental epidemiology [2,46], is a completely comparable tool to thermal indices in assessing heat-related mortality. However, insignificant (and substantially smaller compared to the other indices) cold-related mortality for T suggests that studies based on air temperature (including [21]) may be biased towards too-small estimates of cold effects. This finding is particularly important when the magnitude of changes in heat- and cold-related mortality associated with climate change in temperate regions is estimated [47,48,49]. 

Large differences on cold days as determined by UTCI, with excess mortality in the rural region but no effect in the urban region, suggest that UTCI may be less universal than other indices when applied in bioclimatic and epidemiological analyses (in which “average” thermal conditions for a population are used) as opposed to small-scale biometeorological studies with more specific meteorological input data. However, an influence of complex biometeorological conditions on human thermal comfort is indisputable, and human thermal comfort indices represent the thermal environment better than do simple empirical indices when proper input data are available [20]. Nevertheless, human thermal comfort indices refer to an “average” healthy person, while the population groups most affected by thermal stress are elderly, young children and persons with impaired thermoregulation due to poor physical and medical condition [1,2,50,51]. According to Burkart *et al.* [13], the crucial question for the applicability of human thermal comfort indices to assessing epidemiological outcomes is the significance of the relationship between human health outcomes and the human heat balance. Moreover, the determination accuracy of human thermal comfort indices is affected by uncertainties in modelling mean radiant temperature (Tmrt). If all radiative fluxes are modelled based on synoptic observations (air temperature, air humidity, wind speed and cloudiness), the UTCI’s uncertainties, which are due to uncertainties of the four meteorological input variables, may be as much as 6 °C [52]. This may cause inaccuracy in the thermal stress determination.

In addition to the aforementioned uncertainties in modelling of Tmrt, some other limitations need to be mentioned. Lacking appropriate data, we did not take into account demographic, socioeconomic and other environmental factors (e.g., air pollution) which are significant modifiers of weather-related mortality and should be considered in future research [53,54,55]. Another limitation of this study is the use of airport station data in the analysis for the urban region. The station is situated at the airport on the north-western edge of Prague, which is colder and windier than the city centre, and hence the meteorological data may not be fully representative for the Prague population. Airport stations are often used in similar studies, however, and an analogous dataset from another Prague station was not available. Finally, since the lagged cold effect on mortality is still not wholly explained [28,56,57,58], we did not focus on analyzing lagged effects in this study. This issue needs to be elaborated in follow-up research. 

## 5. Conclusions

We investigated the ability of UTCI and other thermal indices to identify discomfort days having adverse effects on patients with CVD in two regions of the Czech Republic. The results bring new insight to cold-related mortality assessment under temperate climatic conditions and to the applicability of thermal indices for estimating heat and cold effects in populations living in different environments (urban *vs*. rural).

While similar heat effects for air temperature and thermal indices were found in both regions, differences in cold effects between individual indicators were much larger. In particular, UTCI selects windy winter days over the most freezing ones. That results in a small effect on excess mortality in the urban population that is sheltered from the effects of wind and, by contrast, the largest effect (among the examined indices) on excess mortality in the rural population. These findings raise also a critical issue as to the representativeness of wind speed measurements (taken at 10 m height above the surface and strongly determined by local conditions at the measuring site) for estimating human thermal discomfort, particularly in winter. While air temperature seems to be an appropriate tool for heat-related mortality assessment, it appears to be unsuitable when effect of cold on epidemiological outcomes is considered, and thermal indices (PET, AT) yield higher and probably more realistic cold-related mortality.

A universal indicator of human thermal comfort for various related disciplines (biometeorological forecasting, epidemiology, urban and regional planning, bioclimatic mapping, *etc.*) is desirable for easier comparison of results from different geographical areas and on different temporal and spatial scales. UTCI has the potential to become such a useful tool in human biometeorology [4,20]. However, AT (requiring only standard meteorological data) and PET appear to be more universal indicators in heat- and cold-related mortality assessments. Such findings need to be further investigated for other regions and populations, and they are important for determining the final procedure for cold exposure assessment within the UTCI calculation [4,59].
